# Utilization of the Discrete Differential Evolution for Optimization in Multidimensional Point Clouds

**DOI:** 10.1155/2016/6329530

**Published:** 2016-11-15

**Authors:** Vojtěch Uher, Petr Gajdoš, Michal Radecký, Václav Snášel

**Affiliations:** Department of Computer Science and National Supercomputing Center, VŠB-Technical University of Ostrava, Ostrava, Czech Republic

## Abstract

The Differential Evolution (DE) is a widely used bioinspired optimization algorithm developed by Storn and Price. It is popular for its simplicity and robustness. This algorithm was primarily designed for real-valued problems and continuous functions, but several modified versions optimizing both integer and discrete-valued problems have been developed. The discrete-coded DE has been mostly used for combinatorial problems in a set of enumerative variants. However, the DE has a great potential in the spatial data analysis and pattern recognition. This paper formulates the problem as a search of a combination of distinct vertices which meet the specified conditions. It proposes a novel approach called the Multidimensional Discrete Differential Evolution (MDDE) applying the principle of the discrete-coded DE in discrete point clouds (PCs). The paper examines the local searching abilities of the MDDE and its convergence to the global optimum in the PCs. The multidimensional discrete vertices cannot be simply ordered to get a convenient course of the discrete data, which is crucial for good convergence of a population. A novel mutation operator utilizing linear ordering of spatial data based on the space filling curves is introduced. The algorithm is tested on several spatial datasets and optimization problems. The experiments show that the MDDE is an efficient and fast method for discrete optimizations in the multidimensional point clouds.

## 1. Introduction

The Differential Evolution (DE) has been successfully applied to many continuous, combinatorial, and design optimization problems. The measuring devices, cameras, laser devices, or sensors produce discrete multidimensional vertices [1–3]. The big spatial data is analyzed in research areas like robotics, pattern recognition, and/or computer vision. In most of these areas, good results have been achieved with the DE (see, e.g., [1, 4–7]). This paper proposes a novel DE based algorithm solving the combinatorial tasks with discrete vertices. The abilities of the discrete Differential Evolution to search the optimal combinatorial solutions in the multidimensional discrete point clouds (PCs) are discussed. Our modified method called the Multidimensional Discrete Differential Evolution (MDDE) uses a vertex hashing function to strengthen the local properties of an* n*-dimensional discrete dataset.

The Differential Evolution has been introduced by Storn and Price [8]. It is an evolutionary method, which has become popular for its simplicity, robustness, and good convergence properties [9]. It is based on the population of individuals which represent the temporary solutions that are iteratively refined during the generations. Each individual consists of several variables. The quality of individuals is evaluated by an objective function. After the successful DE application for real-valued problems on a continuous space, some combinational or design optimization applications on integer or discrete-valued problems were presented, such as load dispatch problem [10], unit commitment problem [11], 0-1 knapsack problem [12], generalized traveling salesman problem [13], different NP-hard scheduling problems [14–18], form-finding of tensegrity structures [19], assembly line balancing problem [20], or robots path planning problem [21, 22]. Survey of discrete-valued problems and applications of evolutionary algorithms was published in the papers [23–25].

There are several basic categories of variables according to the paper [26]. The discrete integer variables bounded within a range are primarily discussed in this paper. We will call this category the discrete-valued variables. The value of such a variable is an integer pointer addressing an enumerative sample from the set of discrete elements. The elements should be arranged to get better convergence of population [9]; otherwise the DE leads to the random search. The existing discrete methods can be divided into (a) indirect and (b) direct methods. The indirect methods operate with the standard real-valued variables. The values are progressively recalculated to/from the integer ones by some transformation function (see, e.g., [27–30]). The direct methods operate directly with integer values without any transformation, which eliminates the rounding error. The advantages of the indirect methods are that they utilize the robustness of the real-coded DE and require minimal intervention to the original DE. In the paper by Lampinen and Zelinka [27], a simple truncation of the real-valued parameters is proposed. But this simple approach worsens the diversity of the population and the robustness of the algorithm [31]. Other methods using improved rounding techniques involving some additional conditions, constraints, and thresholds were published by Angira and Babu [32], Liao [26], or Schmidt and Thierauf [33]. Tasgetiren et al. introduced several approaches using a discrete DE algorithm for a flow shop scheduling problem [14, 34]. A novel indirect method called the Discrete Differential Evolution (DDE) [29] was proposed by Davendra and Onwubolu. In this case, the whole evolution is managed with the integer values that are transformed into the real ones only for the mutation phase of the DE. This approach uses the Forward Backward Transformation and it has better convergence properties than the simple real/integer rounding techniques [29]. Datta and Figueira described a new mutation operator for discrete-valued variables [35]. Their approach called the ridDE is a direct method based on a bit mutation of integer values to avoid the real/integer transformation.

This paper primarily aims at the problems addressing the optimizations in the sparse discrete data represented by distributed vertices in a vector space. The Differential Evolution is often used for the pattern recognition [7, 36], clustering [37], classification, or feature extraction [38]. All these disciplines find utilization in the bioinspired systems and robot automation [4, 5, 22] or computer vision [36, 39]. The article [38] summarizes different applications of evolutionary algorithms in the pattern recognition and machine learning including the Differential Evolution. The DE has been utilized for human body pose estimation from the point clouds [6, 36, 40], circles detection [7], ellipses detection [41], recognition of leukocytes in images, or 3D face model reconstruction utilizing multiview 2D images [42]. Most of the referenced algorithms optimize analytically a temporary pattern shape, deformable or active shape models. The intersection rate between the proposed model and vertices represents the quality of a solution. However, this means complete passage of whole dataset every time when a solution is evaluated by the objective function. Our further vision is to apply our novel approach to the direct pattern or feature recognition, where an optimized set of discrete vertices represents the required pattern or its estimate.

To do so, some modifications have to be done in the discrete-coded DE. This paper conducts the basic model of the MDDE. The multidimensional vertices are numbered by their indices in the memory. The discrete-valued variables of individuals store the integer indices addressing the vertices in the memory. Thus, the stochastic optimization iteratively refines the vertex indices to find the required combination of vertices. The local searching abilities of the MDDE in the static point clouds are examined. The DE can efficiently handle a nonlinear and a nondifferentiable objective function. Thus, it is expected that it should be applicable to the global optimization problems in the sparse point clouds as well. The main problem is that the discrete vertices are unordered and the optimization is very slow and unstable [9]. The 1D enumerative datasets can be ordered by their values. But in the multidimensional space it is necessary to define some hashing function for the* n*-dimensional vertices. The three space filling curves (SFCs) are tested for the vertex hashing to obtain partly sequenced spatial data (Section 2.2).

First, the used real-coded Differential Evolution and the selected SFCs are introduced in Section 2.  Section 3 describes the whole method, its input parameters, and the utilization of the SFCs. It also solves the problem of duplicate indices generated during the evolution.  Section 4 tests the proposed method on several optimization problems and datasets. It proves that our novel MDDE efficiently works in the spatial discrete data and the more sophisticated SFCs considerably improve the convergence of a population.

## 2. Related Work

First, the reference model of the Differential Evolution is reminded (Section 2.1). Next, several types of the space filling curves (SFCs) are mentioned in Section 2.2.

### 2.1. Real-Coded Differential Evolution

The first Differential Evolution algorithm was presented by Storn and Price in 1995 [43] and then improved in 1997 [8]. It is a simple evolution strategy for a global optimization problem [8, 43]. The paper [44] defines several variants of the DE, but the DE/best/l/bin variant is explained here, because it provides better results for most of the tested optimization problems. The basic algorithm is briefly described as follows.

A population consists of *P* individuals representing the potential solutions of the selected optimization problem. The objective function *f*(*X*) evaluating the quality (objective value) of an individual is defined as *f*(*X*) : *R*_*k*_ → *R*. An individual consists of the *k* real-valued variables that are represented by a vector *X* = (*x*_1_,…, *x*_*k*_). The problem dependent constraints defining the search-space limiting the values of the variables can be established as well [26, 45, 46]. Mostly, the minimal value min(*f*(*X*)) is searched. The process of the evolution is done by generating a new population of individuals with improved objective values. The normalized objective value is usually called the fitness value. The number of generations is limited and labeled as *g*. The individual with the minimum objective value found during *g* generations is returned as the result of the optimization. The appropriate setup of the DE input parameters is discussed in [8, 44]. The process of the DE/best/l/bin algorithm can be described as follows:

(1) At the beginning of the DE, the random population respecting defined constraints is generated.
(2) *x*_*i*,*j*_^*G*^ is a variable value of an individual from an actual population, where *i* ∈ [1, *P*], *j* ∈ [1, *k*], and *G* is a generation counter *G* ∈ [1, *g*].
(3) For *i* = 1,…, *P* of generation *G*,
  (a) different individuals *X*_*A*_^*G*^ and *X*_*B*_^*G*^ are selected in the population randomly, where *i* ≠ *A* ≠ *B*, and the third one is *X*_best_^*G*^, which represents the best known solution so far;
  (b) a mutant vector is computed by the mutation operator: *v*_*i*,*j*_^*G*^ = *x*_best,*j*_^*G*^ + *F* · (*x*_*A*,*j*_^*G*^ − *x*_*B*,*j*_^*G*^), where *F* ∈ [0,2] is the mutational factor and *j* = 1,…, *k*;
  (c) a new individual is computed from the mutant vector by the crossover operator: *u*_*i*,*j*_^*G*^ = *v*_*i*,*j*_^*G*^ if *r*_*i*,*j*_^*G*^ ≤ *C* or *j* = *D*_*i*_^*G*^; otherwise, *u*_*i*,*j*_^*G*^ = *x*_*i*,*j*_^*G*^, where *j* = 1,…, *k*, *C* is the crossover constant (*C* ∈ [0,1]), *r*_*i*,*j*_^*G*^ is a random number (*r*_*i*,*j*_^*G*^ ∈ [0,1]), and *D*_*i*_^*G*^ is a randomly chosen index of individual variable;
  (d) *X*_*i*_^(*G* + 1)^ = *U*_*i*_^*G*^ if *f*(*U*_*i*_^*G*^) ≤ *f*(*X*_*i*_^*G*^); otherwise, *X*_*i*_^(*G* + 1)^ = *X*_*i*_^*G*^, where *X*_*i*_^(*G* + 1)^ is an individual of the next population, *X*_*i*_^*G*^ is an individual of the actual population, and *U*_*i*_^*G*^ is a new proposed individual.
(4) Step (3) is repeated *g* times.

### 2.2. Space Filling Curves

**Figure 1:**
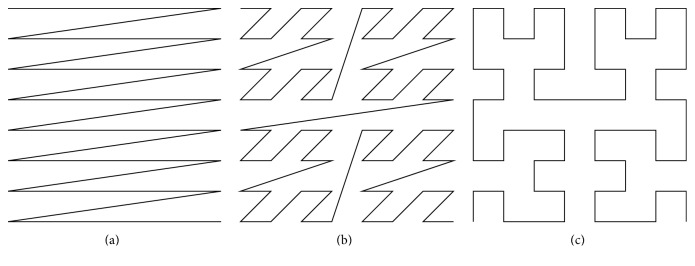
The basic types of the space filling curves ((a) C-curve, (b) Z-order, and (c) Hilbert).

The algorithm proposed in this paper uses space filling curves (SFCs) to represent the multidimensional discrete data. Three variants of the SFCs were selected: linear indexing (C-curve), Z-order, and Hilbert curve (see, e.g., [47, 48] and Figure 1). Generally, SFCs connect the points that are close to each other in the space and thus transform a general* n*-dimensional problem into one dimensional (1D). Any SFC is usually based on a bounded space division. The bounding box of the dataset is computed. For each vertex a code representing its location in the subspace hierarchy is computed, and the vertices are sorted according to these codes. Thus, the ordered linear array grouping the discrete vertices with a similar space character is created. All the three mentioned SFCs are based on the Octree structure, so that they are universally applicable for the* n*-dimensional space. The construction of the SFCs is described in [49, 50]. The SFCs are very straightforward and efficient methods for sparse space clustering [51]. The C-curve is the basic approach for the linearization of the* n*-dimensional data. It can be simply constructed, but the local properties are very basic in comparison with the other two SFCs. The Z-order curve is a very popular curve with good local properties and fast construction times. The Hilbert curve fills the space conveniently without any unnecessary crossings or space leaps (see Figure 1), and thus it is considered to be one of the best Octree based SFCs (see [49, 51]).

## 3. Discrete Differential Evolution in *N*-Dimensional Space

This section describes a novel approach based on the DE for the discrete multidimensional data analysis. The method is explained on the DE/best/l/bin variant (described in Section 2.1), because it seems to be efficient for distance function minimization, but any other variant can be used [44]. The two discrete-coded methods were tested with spatial data: DDE by Davendra and Onwubolu [29] and ridDE by Datta and Figueira [35]. However, the ridDE cannot be parametrized conveniently; thus the DDE was selected as the reference model, as it is introduced in Section 3.1. The problem of discrete vertex optimization is described in Section 3.2. The Multidimensional Discrete Differential Evolution (MDDE) utilized for the *k* distinct solutions search in spatial data is explained in detail in Section 3.3.

### 3.1. Utilized Discrete Model of the DE

The DDE by Davendra and Onwubolu [29] was selected as the reference discrete model, because it works with individuals that consist of the discrete-valued variables. The internal crossover and mutation operators invariably change any applied value to a real number. This leads to infeasible solutions. Therefore, it is necessary to progressively convert the values from integers to real ones and then back to the integers. The DDE uses the so-called Forward Backward Transformation of values. The Forward (integer/real) Transformation is computed only for the mutation and crossover phases of the DE, so that the operators are applied to the real values. The variable values of the new individual are then transformed back to the integers by the Backward (real/integer) Transformation and the evolution continues with the integer values. This model is very convenient for combinatorial problems, where the real values make no sense, and for the detection and elimination of the found duplicate values of an individual. The individual is represented by a vector *X* = (*x*_1_,…, *x*_*k*_). The Forward Transformation is defined as

(1) $$\begin{array}{c} {\left(\;x_{j}\;\right)}^{\prime }=-1+\frac{x_{j}\cdot 500}{999}. \end{array}$$

The Backward Transformation is defined as

(2) $$\begin{array}{c} x_{j}=\left(\;INT\;\right)\frac{\left({\left(x_{j}\right)}^{\prime }+1\right)\cdot 999}{500}, \end{array}$$

where *x*_*j*_ is an integer value and (*x*_*j*_)′ is the corresponding real value for *j* = 1,…, *k*. The constants were established after extensive experimentation [29]. The transformations (1) and (2) are mutually inverse.

### 3.2. Direct MDDE

The modified Multidimensional Discrete Differential Evolution (MDDE) is very similar to the DE from Section 2.1. The most important differences are in the mutation and the evaluation parts. The MDDE optimizes a set of indices addressing the static vertices of the dataset. The vertices are stored in a linear array in the memory. An individual consists of *k* discrete-valued variables. The final solution is defined as a combination of indices addressing the vertices meeting the required conditions. The conditions depend on a specific optimization problem. The objective function can be formulated as a distance function defining some vertex distribution representing, for example, the outline of a required shape.

The main problem is that the real discrete datasets are nonuniformly distributed in the space. Thus, the indices addressing the vertices in the array represent no information about the spatial character of the vertices. Application of the DDE model to the set of unordered vertices leads to the random search. The dataset has to be ordered to get better convergence of the population. However, this is not that straightforward in the* n*-dimensional space; thus smart vertex hashing has to be applied. The three space filling curves are tested in this paper (Section 2.2). SFC makes the* n*-dimensional discrete data partly sequenced, so that the close indices address the spatially close vertices. The specific vertex order affects the diversity of the population and the robustness of the algorithm (see Section 4). The order of vertices is primarily important for the mutational phase of the evolution.

As the MDDE is a randomized algorithm, it is possible that a new generated individual contains some duplicate indices. Generally, a resulting solution consisting of distinct vertex indices is expected to obtain the set of vertices representing the searched pattern or feature. The duplicities have to be eliminated to obtain the duplicity free individuals at the end of every generation. The basic algorithm works as follows:

(1) The input parameters and data are set.
(2) SFC representation of a point cloud is computed.
(3) The initial population of *P* individuals is generated. Each individual consists of *k* discrete-valued variables, which are randomly initialized, so that there are no duplicities.
(4) All individuals are evaluated by the objective function.
(5) For each individual of a population,
  (a) three different individuals are randomly selected from the current population;
  (b) the best known individual and the two of the randomly selected individuals are combined:
    (i) the Forward Transformation (1) of the variable values is computed for all parent individuals;
    (ii) the mutation operator and the crossover operator are applied to the corresponding variables;
    (iii) the variable values of the new individual are transformed to the integers by the Backward Transformation (2) and validated afterwards;
  (c) the duplicate variable values of the new individual have to be resolved; the duplicities are replaced by distinct values from the third randomly selected individual;
  (d) an individual is evaluated by the objective function according to the total objective value (e.g., sum of separate distances); the new individual is compared with the corresponding one from the current population and the better one is selected for the new population;
  (e) the best known solution is compared with the new individual and replaced eventually.
(6) Point (5) is repeated in each of *g* generations.
(7) Finally, the resulting vertices are read according to the found integer indices stored in the discrete-valued variables of the best found individual.

### 3.3. The *k* Distinct Solutions Search

This section describes the parts of the MDDE algorithm in more detail. The utilization of the SFCs, the mutation, and the duplicity elimination are explained here.

#### 3.3.1. Initialization

The input parameters of the MDDE are almost the same as those mentioned in Section 2.1:

 
*l*: number of vertices in the dataset
 
*f*(*X*): total objective function, where *X* = (*x*_1_,…, *x*_*k*_)
 
*f*_*s*_(*x*_*i*_): separate objective function
 
*P*: number of individuals of a population
 
*k*: number of individual variables
 
*n*: dimension of the discrete vertices and the separate objective function
 
*g*: maximum number of generations
 
*F*: constant mutational factor, *F* ∈ [0,2]
 
*C*: crossover constant, *C* ∈ [0,1]

#### 3.3.2. Individual Representation

Each individual of the population consists of *k* discrete-valued variables storing the vertex indices. One array containing the 2*P* individuals is allocated. The alternation of populations is done by the double buffering of *P* individuals and the populations are switched simply by the exchange of pointers addressing the 0th and the *P*th individual. The individual variables are aligned in the memory as well; thus 2 · *P* · *k* values (32-bit) are stored in a row.

#### 3.3.3. Initial Population

The first duplicity free population has to be generated. The range of the vertex indices 〈0, *l* is divided into *k* · *P* blocks. One random index is selected from each block; thus the *k* different initial values are generated randomly for each of *P* individuals. A random permutation of the values is computed afterwards. Therefore, the variable values of all individuals are completely distinguished.

#### 3.3.4. Evaluation

The evaluation of the objective function with an individual is done similarly as it is in the case of casual 1D discrete data. The whole MDDE works with vertex indices assigned by the SFC. The separate objective function is called with a vertex addressed by the integer index. If an individual consists of more variables, a multidimensional objective function will be utilized. Generally, the variables are evaluated by a separate objective function and the sum of *k* particular objective values is used to compute the total objective value of an individual. However, this can be done only if the particular objective value converges by itself (e.g., Euclidean distance). Otherwise, a sophisticated objective function must be used.

#### 3.3.5. Mutation Operator

**Figure 2:**
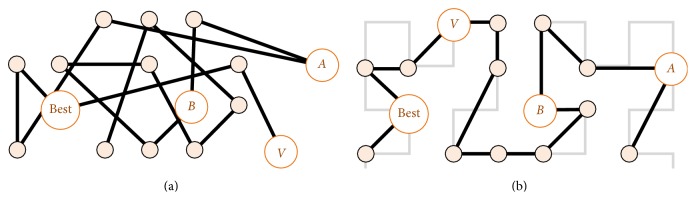
The random vertices (a) are ordered by the Hilbert curve (b). A mutant individual *V* is computed by the mutation operator from the three parent individuals (best, *A*, *B*). The mutation is computed on the level of vertex indices assigned by the corresponding SFC. The vertex order defined by the SFC improves the spatial convergence of the evolution.

The MDDE operates with vertex indices addressing the ordered vertices on the SFC (Figure 2). The mutation operator computes a mutant vector as a linear combination of three different individuals (Section 2.1): two from the current population and the best known one (see Figure 2). According to the DDE model, the mutation operator already calculates with the transformed real values. The computation of the mutant vector is done for each individual variable:

(3) $$\begin{array}{c} v_{i,j}^{G}=x_{best,j}^{G}+F\cdot \left(\;x_{A,j}^{G}-x_{B,j}^{G}\;\right), \end{array}$$

where *i* = 1,…, *P*, *j* = 1,…, *k*, and *G* is a generation counter. Obviously, the mutation operator can be simply reformulated to, for example, DE/rand/l/bin and other variants [44] if it is needed. Figure 2 shows that the order of vertices is crucial for the convergence of population. The SFC better secures that the mutant index *v*_*i*,*j*_^*G*^ computed from the parent indices (3) addresses the vertex that is placed nearby the vertices addressed by the parent indices *x*_best,*j*_^*G*^, *x*_*A*,*j*_^*G*^, and *x*_*B*,*j*_^*G*^. In the case of unordered point clouds, the mutation would practically lead to a random selection of a mutant vector without any spatial logic (see Figure 2).

#### 3.3.6. Crossover Operator

The traditional crossover operator described in Section 2.1 is applied. A proposed (mutant) solution is accepted with the probability *C*. If *k* > 1, the operator will be applied separately for each variable. The variable values of the new individual are transformed to the integers by (2). Additional constraints and the condition that the values (indices) belong to 〈0, *l* have to be validated afterwards. If a variable value is placed out of the interval, a random value in the interval 〈0, *l* will be selected.

#### 3.3.7. Separate Selection Operator

If *k* > 1 and it is possible to assess the quality of the variable values separately, the selection can be made on the level of separate variables. This pretends the average results generated by the simple optimization of the sum of values and improves the convergence of the population. For example, the vertex distance from the proposed pattern can be used as a separate metric.

#### 3.3.8. Elimination of Duplicities

The various combinations of distinct variable values (vertex indices) may lead to the same resulting value due to the convergence to the global optimum. The duplicities have to be found and replaced to get better diversity of a discrete solution. A point cloud is a finite set of vertices; thus a subset of *k* sufficient vertices can fulfil a condition resulting in some pattern or feature recognition. Therefore, the duplicity free solutions are required. All individuals are checked for duplicities before the final individual selection to preserve this demand for the new population.

For each newly generated individual *U*_*a*_^*G*^ another one *X*_*b*_^*G*^ is randomly selected from the current population (the new one is not finished yet), where *G* is a generation number and *a* ≠ *b*. A new individual *U*_*a*_^*G*^ is checked for duplicities at first and the number of recurrences *r* is obtained, where *r* ∈ 〈0, *k*. The mentioned facts mean that *U*_*a*_^*G*^ and *X*_*b*_^*G*^ can have maximally *k* − *r* identical indices after the elimination of *r* recurrences from *U*_*a*_^*G*^. Thus, having the certainty of the duplicity free *X*_*b*_^*G*^, the remaining *r* indices can replace the recurrences of *U*_*a*_^*G*^.

**Figure 3:**
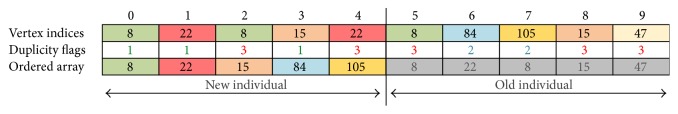
The table shows the copies of two individuals with *k* = 5. The new proposed individual contains two duplicities (indices 8 and 22). They will be replaced with the distinct indices of a selected individual from the old population. The unique indices of the new individual are flagged by 1, by 2 in the case of the old individual. The duplicities and the excess indices are flagged by 3. The sorting of indices by flags produces a new duplicity free individual.

The implementation of this algorithm is based on convenient flagging of the indices followed by their sorting (Figure 3). The index arrays of both individuals are copied to the temporary array one by one. *U*_*a*_^*G*^ is stored at first followed by *X*_*b*_^*G*^. Another array holds the corresponding flags of 2*k* indices. The flagging is done by the sequential comparison of unmarked indices. The indices of *U*_*a*_^*G*^ are flagged at first and the *r* recurrences are found. The unique indices are flagged with 1 and the duplicities with 3. Next, the *r* distinct indices have to be found in *X*_*b*_^*G*^, so that the indices of *X*_*b*_^*G*^ are compared with the preceding ones. The unique indices are flagged with 2 and the search will be terminated when *r* indices are found. The remaining indices are flagged by 3. The 2*k* indices are sorted by Quick sort algorithm according to the flags; thus the first *k* indices represent the new duplicity free individual. The flagging can be also used for penalization of undesired solutions so that the penalized indices are sorted out.

#### 3.3.9. Final Remark

The new proposed individual is compared with the best known one. The total objective value is used to assess the best ascertained individual. The whole computation is terminated after *g* generations, or when a terminating condition is met. The ascertained individual with the best total objective value is returned.

## 4. Experiments and Discussion

In this section, the proposed MDDE method is tested. The main aim of the experiments is to test the local behaviour of the MDDE on the three space filling curves (SFCs) and its convergence to the global optimum in the discrete point clouds (PCs). The C-curve was selected as a naive vertex hashing algorithm for comparison to show that the MDDE running on more complex SFCs with better local properties converges faster to the searched extreme. It seems there is no comparable method addressing the combinational problems on the level of discrete multidimensional vertices. The SFCs are constructed by hierarchical vertex hashing followed by sorting of the vertices according to the hashes/codes (see Section 2.2). A code represents an octant that contains the hashed vertex. The order of the octant written to the code distinguishes the different variants of the SFCs. The codes are usually represented by a bit sequence of octant coordinates. The SFCs of all the tests and datasets were constructed for the maximum hierarchical level allowed by the 64-bit integer. The bit length of the hash is the main limitation of our method, because the greater the dimension *n* of the discrete vertices is the lower the maximum level of clustering and the ability of the SFCs to distinguish location of two close vertices are. That is why the experiments are focused on 2D and 3D problems and datasets. But the MDDE is generally applicable for* n*-dimensional spaces if the longer hashes are used.

This paper primarily aims at the problems addressing the optimizations in the sparse discrete data represented by distributed vertices in a vector space. It is assumed that the observed property or the pattern is locally bound to the spatial data. Several discrete methods were tested, but the DDE by Davendra and Onwubolu [29] has been chosen. In comparison with the ridDE [35], the DDE provides the option of the *F* parameter setting that allows one to define the sampling step of the evolution. All the tests were performed with the DE/best/l/bin variant, as this seems to be the best one after extensive experimentation.

### 4.1. The Definition of the Tested Problems

The algorithm was tested on several common optimization problems:

1. point-to-point and point-to-line distance minimization problem
2. discrete optimization of Schwefel and Rastrigin functions
3. maximum distance search in 3D datasets

These problems have been selected, because they are applicable for all kinds of point clouds and space dimensions and they mostly represent the basic tasks in the area of the spatial data analysis. They can be precisely solved analytically by the brute force vertex comparison as well; thus it is possible to compare the results of the analytical and the evolutionary approaches. The problems are described in the following subsections.

#### 4.1.1. Point-to-Point and Point-to-Line Distance Minimization

The objective function of the point-to-point problem is defined as the Euclidean distance between a randomly chosen vertex $\vec{p}$ from the dataset and the vertices proposed by an evolution. The objective function of the point-to-line problem is defined as the Euclidean distance between the line constructed by two different vertices randomly chosen from the dataset and the vertices proposed by an evolution [52]. The distance is the basic metric that is generally minimized to recognize some shape or pattern. The evolution converges locally to the global extreme in this case; thus it is a good example that can be tested with the MDDE. It is obvious that the randomly selected vertices have to be consistent during the whole evolution process. The distances of the *k* vertices of each individual are optimized separately and the total fitness (objective) value of an individual is computed as a sum of *k* distances. In both cases, the zero distance solutions are heavily penalized in order to provide the comparison rating between the analytical and the evolutionary approach.

#### 4.1.2. Discrete Optimizations of Test Functions

The evolutionary algorithms are usually checked on several continuous test functions [53]. The well-known Schwefel and Rastrigin functions have been selected for the tests of the MDDE, because they are both very complex functions with many local minima and they are applicable for any dimensions (see [53]). These continuous functions represent the corresponding objective functions evaluating the quality of the ascertained vertices. The discrete vertices of the dimension *n* are randomly generated in the typical input domains defined, for example, in [53]. Thus the optimization is based on the search of the *k* distinct vertices with the minimal objective value.

**Figure 4:**
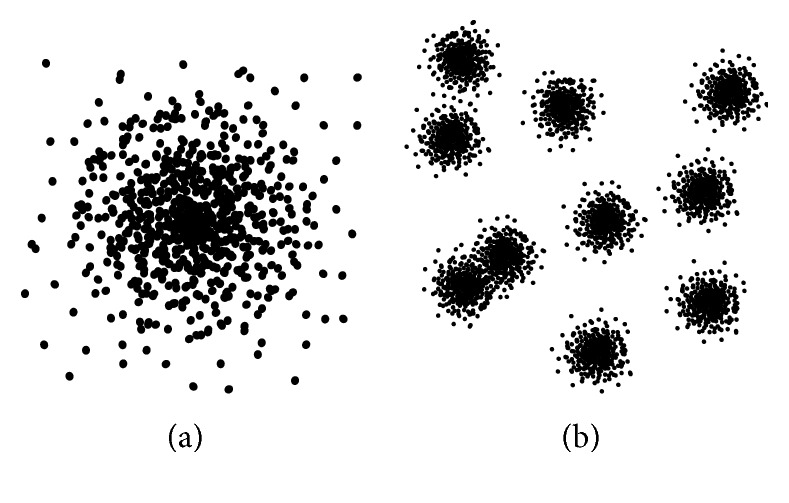
Vertex distributions: (a) Gaussian; (b) Gaussian islands.

Two different distributions of random samples were tested to better distinguish the properties of the space filling curves (see Figure 4). The Gaussian distribution consists of 10^5^ vertices sampled randomly according to the standard normal distribution recalculated to the intervals of the input domain. Similarly, the Gaussian islands are the ten randomly chosen vertex groups distributed according to the standard normal distribution (Figure 4(b)) containing together 10^4^ vertices. The distributions are the same for all measurements.

#### 4.1.3. Maximum Distance Search

The problem is defined as a search of the two most distant vertices of the dataset. This can be used, for example, as an approximative solution of the minimum sphere problem, which is defined as a search of the minimum sphere containing all the vertices of the dataset [54]. The minimum sphere problem is more complex, because the maximum Euclidean distance used as a perimeter of the sphere does not guarantee that all the vertices are contained inside the sphere. However, in many cases the maximum distance can be used as a good estimate of the minimum sphere problem solution, which can be further improved. We reformulated it to a minimization problem, so that the difference

(4) $$\begin{array}{c} \Delta Dist=diagonal-maxDist \end{array}$$

is minimized, where diagonal is the diagonal length of the bounding box and maxDist is the maximum distance between two vertices found in the dataset. The bounding box diagonal represents the possible maximum distance of two vertices; thus ΔDist is always positive.

This problem is different from the others, which locally converge to the extremes. But the maximum distance can be found by the local search of two distant areas, which leads to finding of greater distances. Therefore, the MDDE algorithm converges to the global extreme as well.

### 4.2. Achieved Results

This section discusses the achieved results of the MDDE tested on the defined problems. The three artificial and the three real standard datasets were chosen for the tests, as they are mentioned in Table 1. The random Gaussian datasets were generated according to standard normal distribution. The Gaussian islands were explained in Section 4.1.2. For all optimization problems and datasets the best solutions are computed analytically in advance.

#### 4.2.1. Sufficient Solution Search

**Figure 5:**
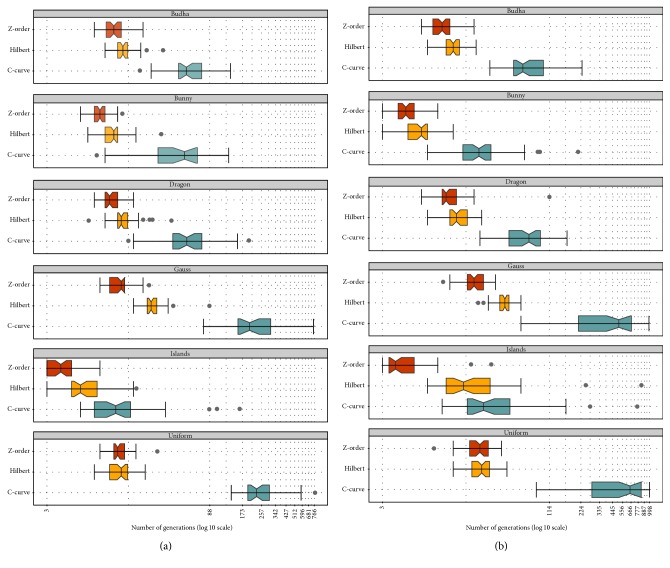
The box plots comparing the SFCs on the* point-to-point* (a) and* point-to-line* (b) distance minimization problems in the 3D space. The horizontal axis shows the number of generations needed to reach the sufficient fitness rate according to Table 1. Each measurement was done 50 times for the same parameters and datasets (see Table 1).

First, the point-to-point and point-to-line problems were tested (see Section 4.1.1). The corresponding DE parameters for both problems can be seen in Table 1 and they were established after extensive experimentation.  Figure 5 shows the comparison of the SFCs on the six different 3D datasets. These tests measure the number of DE generations needed to obtain a sufficient result, so that all *k* vertices must have the sufficient distance. The sufficient result *X*_best_^*G*^ has to meet the condition

(5) $$\begin{array}{c} f_{s}\left(\;x_{best,j}^{G}\;\right)<f_{best}\cdot fitnessRate \end{array}$$

for *j* = 1,…, *k*, where *k* is the number of individual indices, *G* is a generation counter, *f*_*s*_ is a separate objective function that returns the distance of the *j*th individual vertex from the reference point, *f*_best_ is the best analytically computed solution, and fitnessRate is the corresponding accuracy rate according to Table 1. Each measurement was performed 50 times for different randomly selected vertices, which define the reference vertex or line. Thus, the graphs represent the convergence metrics examining various areas of the distributed datasets.  Figure 5 shows that the MDDE utilizing the Z-order and the Hilbert curve converges faster to the global optimum than in the case of the C-curve. The Z-order generally shows better results than the Hilbert curve especially in sparse and nonuniformly distributed datasets.

#### 4.2.2. Convergence Tests

**Figure 6:**
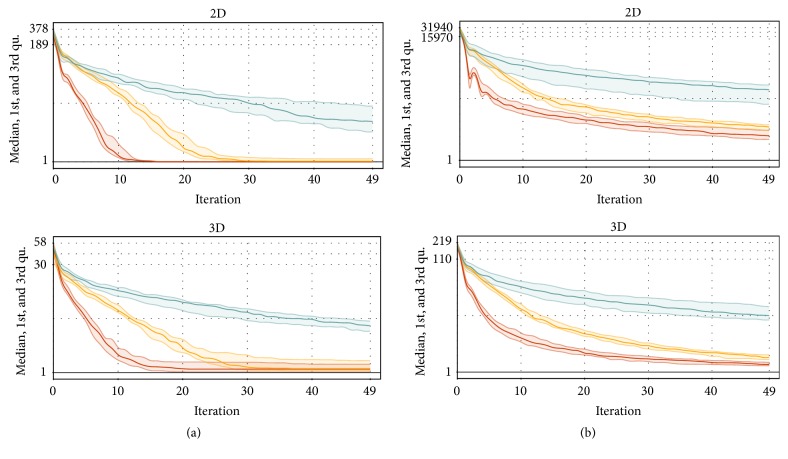
The ribbon plots comparing the evolutions on* point-to-point* (a) and* point-to-line* (b) distance minimization problems for different SFCs (red: Z-order, yellow: Hilbert, and green: C-curve) and artificial datasets with the Gaussian distribution (2-3 dimensions). The vertical axis (log⁡10 scale) shows the fitness value expressed by a multiple of the best solution precomputed analytically. Each measurement was performed 20 times for the same parameters (see Table 1).

**Figure 7:**
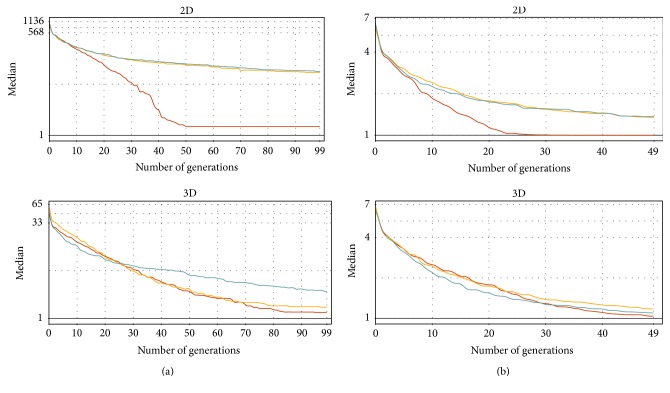
The convergence of the discrete evolution on the* Rastrigin* function utilizing the different SFCs (red: Z-order, yellow: Hilbert, and green: C-curve). The vertical axis (log⁡10 scale) shows the fitness value expressed by a multiple of the best solution precomputed analytically. The measurements were performed 20 times for two different vertex distributions ((a)* Gaussian*; (b)* Gaussian islands*) and the same parameters (Table 2).

**Figure 8:**
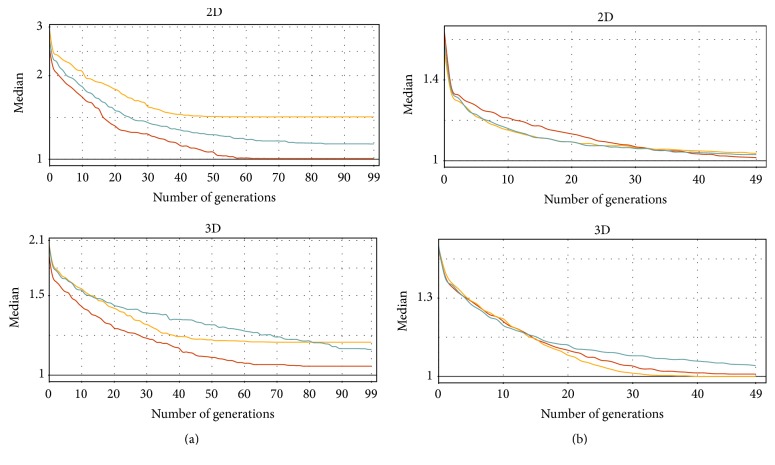
The convergence of the discrete evolution on the* Schwefel* function utilizing the different SFCs (red: Z-order, yellow: Hilbert, and green: C-curve). The vertical axis (log⁡10 scale) shows the fitness value expressed by a multiple of the best solution precomputed analytically. The measurements were performed 20 times for two different vertex distributions ((a)* Gaussian*; (b)* Gaussian islands*) and the same parameters (Table 2).

**Figure 9:**
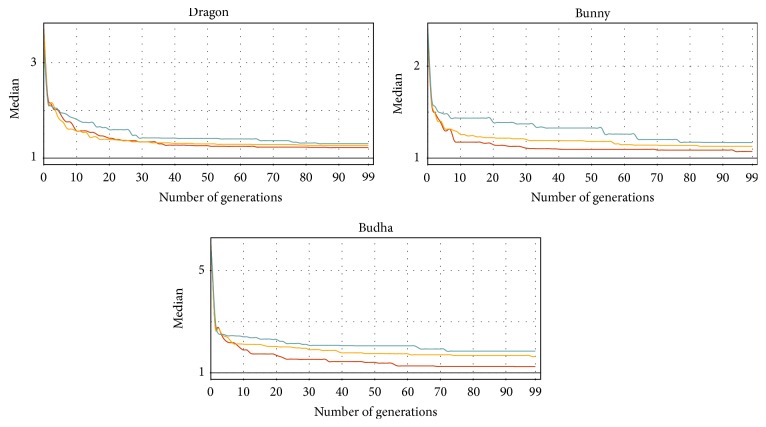
The convergence of the discrete evolution on the* maximum distance search* problem utilizing the different SFCs (red: Z-order, yellow: Hilbert, and green: C-curve). The vertical axis (log⁡10 scale) shows the fitness value expressed by a multiple of the best solution precomputed analytically. The measurements were performed 20 times for 3 real 3D datasets and the same parameters (Table 2).

The next measurements are focused on the evolution convergence during the generations. Figures 6, 7, 8, and 9 show the MDDE progress measured on different problems, datasets, and dimensions. These measurements are visualized by the ribbon plots or curves of medians constructed from 20 preformed measurements. The vertical axis represents the corresponding fitness value expressed by a multiple of the best analytical solution.

Figure 6 shows the comparison of the ribbon plots displaying the median, the first, and the third quartile of the measured fitness for point-to-point and point-to-line problems. These tests were performed on the artificial datasets with 10^6^ vertices with the Gaussian distribution according to the parameters in Table 1. The Z-order shows its supremacy again; the C-curve has the worst convergence in this measurement. The accuracy is much better in the case of the point-to-point distance problem, because the line crosses the whole point cloud; thus there are many very close vertices. The vertices with the zero distance metric are eliminated in both cases.

Figures 7 and 8 show similar convergence metrics for the Rastrigin (Figure 7) and the Schwefel (Figure 8) test functions. Only the medians are displayed to obtain better legibility of the plots.  Table 2 summarizes the MDDE parameters for all tests. The tests on both functions were performed on artificial datasets with the Gaussian distribution (10^5^ vertices) and Gaussian islands (10^4^ vertices), as it was explained in Section 4.1.2. The results are more comparable in contrast with the distance functions especially in the case of the Gaussian islands. However, the Z-order mostly shows the fastest convergence and the best accuracy in comparison with the other SFCs.

Finally, Figure 9 represents the convergence metrics of the maximum distance problem reformulated to the minimization problem (see Section 4.1.3). These tests were performed on the three Stanford datasets mentioned in Table 1 according to the parameters in Table 2. The plots show the progress of the fitness rate during the 100 generations. The results are quite comparable again, but the Z-order converges faster than the Hilbert curve and C-curve.

#### 4.2.3. Completeness Tests

The MDDE returns a vector of vertex indices as a result of the optimization. The discrete optimal solutions can be found analytically in the datasets with the finite number of vertices, so that the intersection of the stochastically found solution and the best solution can be computed. Thus, the completeness is defined by the rate

(6) $$\begin{array}{c} c=\frac{a}{k}, \end{array}$$

where *a* is the number of correctly found vertex indices of an individual and *k* is the total number of individual indices.

The completeness was measured after 100 generations of the evolution on the Rastrigin (Table 3) and the Schwefel (Table 4) test functions, because they are very complex functions with many local minima. The measurements were performed with the DE parameters summarized in Table 2. Tables 3 and 4 represent the completeness comparison for the three SFCs and two vertex distributions. The tables show that the completeness is better in the case of Gaussian islands and 3D space. The same number of vertices distributed in the 2D space leads to the greater density of sampling; thus there are more vertices with good fitness than in the 3D space, where the distances between samples are greater. Therefore, the distinction of two very close solutions is very complicated for such a bioinspired method. However, the results are still very good especially in the case of Z-order and Hilbert curves.

#### 4.2.4. Performance Tests

This section briefly introduces the performance of the proposed MDDE algorithm. The evolutionary times of 100 generations including the duplicity elimination are summarized in Table 5. Each measurement was performed 50 times on all the mentioned optimization problems according to the DE parameters in Tables 1 and 2. The computation times basically rely on a population size *P*, the number of individual variables *k*, the dimension *n*, and the objective function. All experiments run on the following hardware: Intel Core i5 760 @ 2.8 GHz, 16 GB RAM, Windows 7 64-bit.

## 5. Conclusion

A novel modification of the DE called the Multidimensional Discrete Differential Evolution (MDDE) addressing the combinatorial problems in* n*-dimensional point clouds is presented. Our method aims at the discrete-valued problems, where a combination of multidimensional vertices represents the required solution. The convergence of the evolution is improved by spatial data linearization by the space filling curves (SFCs). The algorithm efficiently eliminates the problem of the duplicate values in an individual. The paper examines the local searching abilities of the MDDE and the convergence to the global extreme in the discrete point clouds. The method is tested on several spatial optimization problems and the three SFCs (Z-order, Hilbert, and C-curve). The tests on the convergence and completeness of the discrete solution show that the Z-order curve can be recommended as the best variant from the tested SFCs. The completeness of the best found solutions mostly balances between 60% and 100% depending on the used SFC. The evolution converges fast especially during the first 50 generations. The computation times of 100 generations measured on the test problems are maximally several milliseconds. Our MDDE is an efficient and fast method for discrete optimizations in the multidimensional point clouds. The main disadvantage of the MDDE is the limited precision of the SFCs, which are limited by the bit length of the vertex hashes. This is considerable especially in higher dimensions.

The MDDE represents a basic discrete model for pattern recognition and feature extraction especially in the 2D and 3D discrete datasets. The difficult task is to formulate the real problems for the MDDE; thus this will be the direction of our future work. We have promising results in the area of primitives detection, where the MDDE can accelerate the convergence of evolution.

## Figures and Tables

**Table 1:** The DE parameters for point-to-point and point-to-line minimization problems and different datasets.

Dataset	Vertex num. (*l*)	Fitness rate	
		PP^*∗*^	PL^*∗*^
Uniform rand.	10^6^	2.0	5.0
Gauss. rand.	10^6^	2.0	5.0
Gauss. islands rand.	10^4^	1.5	5.0
Stanford Bunny^1^	35947	1.8	5.0
Stanford Dragon^1^	437645	2.2	5.0
Stanford Budha^1^	543652	2.2	2.0

Common parameters	*C* = 0.95		
	*k* = 16 (PP^*∗*^) or *k* = 50 (PL^*∗*^)		
	*F* = 0.01 (PP^*∗*^) or *F* = 0.005 (PL^*∗*^)		
	*P* = 30 (PP^*∗*^) or *P* = 80 (PL^*∗*^)		

^*∗*^Point-to-point (PP); point-to-line (PL).

^1^Models from The Stanford 3D Scanning Repository [55].

**Table 2:** The DE parameters for Rastrigin and Schwefel function minimization problem and maximum distance problem for different vertex distributions.

Rastrigin function				
Distribution	*k*	*P*	*F*	*C*
Gaussian 2D	10	30	0.1	0.5
Gaussian 3D	10	30	0.05	0.5
Gaussian islands 2-3D	10	20	0.3	0.5

Schwefel function				
Distribution	*k*	*P*	*F*	*C*

Gaussian 2-3D	10	30	0.05	0.5
Gaussian islands 2-3D	10	20	0.3	0.5

Maximum distance				
Distribution	*k*	*P*	*F*	*C*

Stanford datasets	2	30	0.3	0.8

**Table 3:** Rastrigin function: completeness of results after 100 generations.

Z-order	2D			3D		
Distribution	Avg.	Stdev.	Med.	Avg.	Stdev.	Med.
Gaussian	0.61	0.25	0.5	0.54	0.23	0.6
Gauss. islands	0.94	0.09	1.0	0.90	0.09	0.9

Hilbert	2D			3D		
Distribution	Avg.	Stdev.	Med.	Avg.	Stdev.	Med.

Gaussian	0.04	0.05	0.0	0.67	0.32	0.8
Gauss. islands	0.22	0.12	0.2	0.81	0.08	0.8

C-curve	2D			3D		
Distribution	Avg.	Stdev.	Med.	Avg.	Stdev.	Med.

Gaussian	0.04	0.06	0.0	0.16	0.11	0.2
Gauss. islands	0.21	0.11	0.2	0.93	0.06	0.9

**Table 4:** Schwefel function: completeness of results after 100 generations.

Z-order	2D			3D		
Distribution	Avg.	Stdev.	Med.	Avg.	Stdev.	Med.
Gaussian	0.8	0.24	0.9	0.93	0.13	1.0
Gauss. islands	0.89	0.08	0.9	0.83	0.1	0.8

Hilbert	2D			3D		
Distribution	Avg.	Stdev.	Med.	Avg.	Stdev.	Med.

Gaussian	0.8	0.28	1.0	0.86	0.18	0.9
Gauss. islands	0.7	0.09	0.7	0.95	0.16	1.0

C-curve	2D			3D		
Distribution	Avg.	Stdev.	Med.	Avg.	Stdev.	Med.

Gaussian	0.59	0.15	0.6	0.23	0.14	0.2
Gauss. islands	0.81	0.07	0.8	0.8	0.08	0.8

**Table 5:** Computation times (s) of 100 generations of the defined problems.

Problem	2D			3D		
	Avg.	Stdev.	Med.	Avg.	Stdev.	Med.
Point-to-point	0.0075	0.0005	0.007	0.0077	0.0005	0.008
Point-to-line	0.0653	0.0005	0.065	0.0694	0.0006	0.069
Rastrigin (Gaussian)	0.0059	0.0005	0.006	0.0073	0.0004	0.007
Rastrigin (Gauss. islands)	0.004	0.0004	0.004	0.0044	0.0005	0.004
Schwefel (Gaussian)	0.0067	0.0004	0.007	0.0083	0.0005	0.008
Schwefel (Gauss. islands)	0.0044	0.0005	0.004	0.0053	0.0005	0.005
Maximum distance	—	—	—	0.0019	0.0003	0.002
